# Executive Function After Prenatal Alcohol Exposure in Children in a South African Population: Cross-sectional Study

**DOI:** 10.2196/20658

**Published:** 2021-07-02

**Authors:** Jacobus Gidion Louw, Alastair van Heerden, Leana Olivier, Tersius Lambrechts, Mandi Broodryk, Liska Bunge, Martlé Vosloo, Mark Tomlinson

**Affiliations:** 1 Foundation for Alcohol Related Research Cape Town South Africa; 2 Department of Psychology Stellenbosch University Stellenbosch South Africa; 3 Institute for Life Course Health Research Department of Global Health Faculty of Medicine and Health Sciences, Stellenbosch University Cape Town South Africa; 4 Centre for Community Based Research Human Sciences Research Council Pietermaritzburg South Africa; 5 Developmental Pathways for Health Research Unit Department of Paediatrics, Faculty of Health Science University of the Witwatersrand/Medical Research Council Johannesburg South Africa; 6 School of Nursing and Midwifery Queens University Belfast United Kingdom

**Keywords:** fetal alcohol spectrum disorders, FASD, cognitive, executive function, experimental games, brain drug effects, child development, serious games, games, alcohol, training

## Abstract

**Background:**

Alcohol is a teratogen; its consumption during pregnancy can lead to negative birth outcomes, collectively referred to as fetal alcohol spectrum disorders. Neurodevelopmental delays in higher-order cognitive functions that affect development of executive functions are a common feature. Studies on executive function in children have focused on children diagnosed with fetal alcohol spectrum disorder, and there is a lack of information on the impact on children not diagnosed with fetal alcohol spectrum disorder but who had been exposed to alcohol.

**Objective:**

The aim of this study was to compare the development of executive function in children between 4 and 6 years of age with and without prenatal exposure to alcohol.

**Methods:**

Children both exposed and not exposed to alcohol were recruited as part of a feasibility RCT evaluating a computer-based cognitive training program for improving executive function development. The study was conducted in a low–socioeconomic status community in South Africa with a high prevalence of fetal alcohol spectrum disorder. Neurodevelopment was assessed in participating children; NEPSY-II standardized scores for executive function domains were compared using a multivariate analysis of variance with group membership as the predictor variable.

**Results:**

No significant differences in executive functions assessments (*P*=.39) were found between children in the alcohol-exposed group (n=76) and those in the nonexposed group (n=40). Both groups showed moderate to severe delays in domains. In all but one subtest, the average score for both groups was below the 25th percentile of expected norms.

**Conclusions:**

We expected that alcohol exposure would have a measurable impact on executive function development. The lack of differences highlights the prevalence of developmental delays in low–socioeconomic status communities in South Africa and suggests that children are exposed to various threats to cognitive development.

**International Registered Report Identifier (IRRID):**

RR2-10.2196/14489

## Introduction

### Prenatal Alcohol Exposure

Alcohol is a known teratogen when consumed during pregnancy and can lead to a number of negative outcomes including a characteristic pattern of dysmorphic facial features, growth retardation, deficient brain growth, and neurodevelopmental delays in the newborn child [1,2]. These outcomes vary in their presentation and severity and are grouped as fetal alcohol spectrum disorders. Diagnostic labels are assigned based on the number and pattern of associated characteristics of fetal alcohol spectrum disorder that are present. There are 4 diagnostic categories: fetal alcohol syndrome, partial fetal alcohol syndrome, alcohol-related neurodevelopmental disorder, and alcohol-related birth defects [1,3]. Apart from alcohol-related birth defects, neurodevelopmental delays are a common feature of all diagnoses [1].

The most common form of fetal alcohol spectrum disorder is alcohol-related neurodevelopmental disorder, for which physical features are not evident. As such, it is also the most difficult of the fetal alcohol spectrum disorders to diagnose [4]. The lack of observable physical features does not mean that the neurodevelopmental delays are less pronounced or serious than those in children with fetal alcohol syndrome. Affected individuals can have the full range of cognitive impairments associated with the other diagnoses [1,5,6]. It is important, therefore, to bear in mind that even if a person exposed to alcohol in utero does not meet fetal alcohol spectrum disorder criteria, there can still be negative developmental sequelae.

### Fetal Alcohol Spectrum Disorder and Executive Function

Elementary and higher-order intellectual functions can be affected in individuals with fetal alcohol spectrum disorder, and dysfunction can be present regardless of whether the physical features of fetal alcohol spectrum disorder are present [7]. Some areas of particular concern include general intellectual ability [7-10], memory and learning [8,11,12], adaptive living skills [10], and executive functions [10,13,14].

Deficits in executive function are hallmarks of fetal alcohol spectrum disorder, with far-reaching consequences for affected individuals [7,8,13]. The term *executive functions* refers to a group of cognitive domains involved in guiding thoughts and goal-directed behavior [15]. The 3 main executive functions are inhibitory control, cognitive flexibility, and working memory [16,17], which are required for, among others, inhibiting inappropriate responses, task planning, and emotional regulation. These areas are also required for self-monitoring performance to identify and self-correct errors. Dysfunction in these areas is, therefore, associated with poor academic outcomes, behavioral problems, mental disorders, and difficulties in daily functioning [7,13,16,18].

### Cognitive Training of Executive Function

Executive function in children is amenable to intervention [19-21]. There is considerable evidence that, when specific executive function processes are trained, improvements from training are also evident in similar domains [16,21,22], such as structurally similar cognitive training tasks, which is referred to as *near transfer* [23]. There is also limited evidence for far transfer, which is the transfer of improvements between structurally different tasks or fluid intelligence that depends on executive function [21,22]. Improvements are more pronounced in children with delays in executive function, which has been found in children with fetal alcohol spectrum disorder [16]. Because far transfer is not guaranteed, it is important to target the most significant areas of deficit for training through the intervention. A distinct profile of attention deficits may exist in children with fetal alcohol spectrum disorder, which could highlight where efforts need to be focused [24]. Studies in this field have focused on children who have already been diagnosed with fetal alcohol spectrum disorder [14,20,25]; however, a number of children affected by prenatal alcohol exposure may not meet the criteria for fetal alcohol spectrum disorder diagnosis and may be overlooked in epidemiological studies [26].

### Study Aim and Hypothesis

If a diagnosis of fetal alcohol spectrum disorder is required for inclusion in studies on prenatal alcohol exposure’s effect on executive function, the picture of alcohol’s impact in utero will be incomplete. The aim of this paper was to compare executive function in children who had been exposed with those who had not been exposed to alcohol during prenatal development. Data for the analyses were obtained as part of a feasibility RCT of a computer-based cognitive training game. Assessments of executive function were conducted on children both exposed and nonexposed to alcohol. The primary hypothesis for this paper was that children in the alcohol-exposed group would perform poorer than those in the nonexposed group on standardized measures of executive function.

## Methods

### Design

**Figure 1:**
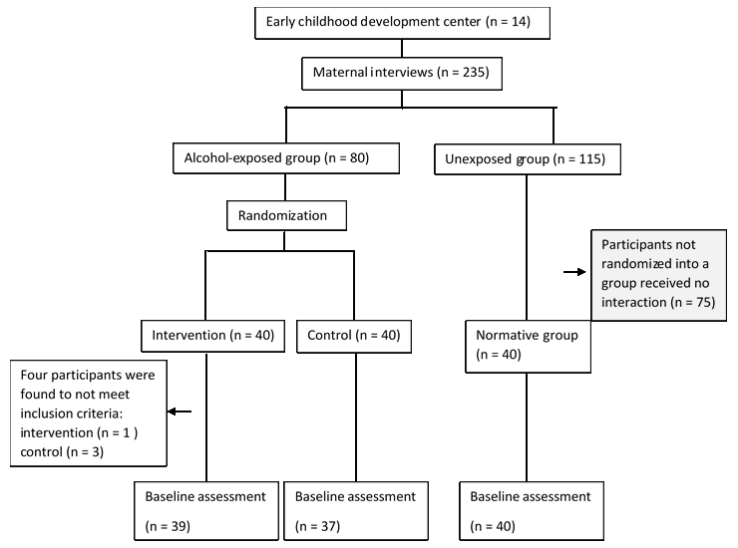
CONSORT diagram.

This paper reports the findings of baseline assessments from a feasibility RCT of a computer-based cognitive training game (International Standard Randomized Controlled Trial Number; ISRTCN17244156). The trial protocol has been published [27]. Assessments were conducted at baseline before the start of the RCT intervention. The RCT comprised 3 arms. Children were recruited from local early childhood development (ECD) centers and assigned to groups based on in utero alcohol exposure, which was identified by interviewing children’s biological mothers (Figure 1). Children exposed to alcohol were randomly assigned 1:1 to either the control or intervention group using block randomization. Once the intervention and control groups had been finalized, 40 unexposed children were individually selected for the third arm using random number tables [28] in order to provide normative data. Baseline assessments were completed for all 3 groups. In line with the sample sizes of previous studies [12,29,30] on fetal alcohol spectrum disorder and cognitive function, a target of 120 participants was set.

### Setting

Saldanha Bay municipality largely comprises Saldanha Bay, a harbor city with a population of 113,239 as of 2017 and main industries of agriculture, forestry, fishing, and manufacturing [31]. There is a high unemployment rate (17%) and significant income inequality (Gini coefficient 0.59 [31]). The prevalence of fetal alcohol spectrum disorder in the municipality is 64 per 1000 individuals [32].

### Participants

Because we were interested in a population of children between the ages of 4 and 6 years, we approached an organization called Saldanha Bay ECD forum that serves ECD centers in low–socioeconomic status areas of the Saldanha Bay municipality. After giving an overview of the RCT, meetings were set up with the individual ECD center principals. At these meetings, the project was discussed in more depth, and the principals were asked if they would be willing to facilitate access to the parents of the children in their schools (ie, inform parents of the study and ask if they would like to participate).

We obtained the contact details of parents willing to allow their children to participate in the study, and community workers contacted these parents, obtained informed consent, and conducted interviews with biological mothers. The order in which parents from participating ECD centers were contacted was determined by randomly allocated numbers using Microsoft Excel. All consenting mothers identified by a particular ECD center were contacted for interviews before we moved on to mothers from another ECD. If, however, there were consenting mothers who were unavailable for interviews, we still moved on to a new ECD center. We followed up to see if they became available for interview at a later stage. New ECD centers were included until 80 participants exposed to alcohol had been identified, following which the 40 unexposed participants were selected.

### Ethical Considerations

Ethics approval was obtained from the Health Research Ethics Committee at Stellenbosch University (N16/05/063). Data were deidentified after participants were allocated study IDs, and test administrators were blinded to alcohol exposure status during assessments.

### Procedure

The structured interview used with the children’s mothers has been used extensively in fetal alcohol spectrum disorder epidemiological studies in South Africa [32-34]. During the interview, a questionnaire was completed which included questions on sociodemographic information, pregnancy behavior, and alcohol use during pregnancy. The interviews were conducted and questionnaires were completed by community workers trained in conducting this interview. Training was provided by one of the authors (JL) and included roleplay and recorded practice interviews. The interviews were conducted at the mothers’ homes.

Children who were exposed to more than 3 standard drinks in 1 drinking session during pregnancy were defined as having had prenatal alcohol exposure.

### Measures

Neurodevelopmental assessments of the children were conducted by trained psychometrists using NEPSY-II, a battery of individually administered tests that has been shown to reliably diagnose a range of childhood disorders [35]. Subtests can be selected based on the domains to be assessed [36]. NEPSY-II has been used in studies on fetal alcohol spectrum disorder and executive function [14,37] and has been extensively used globally, including in low- and middle-income countries [30]. Although no validation study for NEPSY-II has been conducted with data from South Africa, NEPSY-II has been successfully used in South African contexts [38,39]. We selected subtests that assessed attention and executive function; language; and memory and learning (Table 1).

**Table 1:** NEPSY-II domains and subtests.

Domain	Subtest
Attention and executive function	Statue
Language	Comprehension of instructions
Memory and learning	Memory for designs content Memory for designs spatial Memory for designs Narrative memory free and cued Narrative memory and recognition contrasted Sentence repetition

### Data Analysis

Data were collected using REDCap [40] (Vanderbilt University) and analyzed using SPSS statistical software (version 25; IBM Corp). Since RCT group membership was not the variable of interest, to evaluate the impact of alcohol exposure, NEPSY-II scores for children in the alcohol group (RCT intervention and control groups) were compared to those from children in the nonexposed group. Before combining the RCT intervention and control groups, the demographic variables of the 3 groups were compared using 1-way analysis of variance and chi-square tests. The mean and median alcohol exposure for the alcohol-exposed group were also calculated. The mean and median scaled scores for subtests were calculated and compared to norms [35]. The scores were interpreted using the NEPSY II–suggested classification labels [41].

To evaluate whether alcohol exposure predicted test performance, multivariate analysis of variance with group membership as the predictor variable was conducted to test the scaled scores of the 8 subtests. A Box *M* test for homogeneity of covariance was not significant (*P*=.59). The absence of multicollinearity was assumed for this analysis. Due to possible violation of some of the assumptions for multivariate analysis of variance, we used Pillai trace as the test statistic with a conservative level of α=.01.

## Results

After engaging with the ECD forum, 27 ECD centers agreed to participate. To obtain 80 children with prenatal alcohol exposure, 235 interviews were completed in 14 ECD centers. Upon review of the assessment data, 4 children were excluded for being too young, and their data were excluded from analysis. The final sample (n=116) included 76 children in the alcohol-exposed group and 40 children in the nonexposed group (Figure 1).

There was a significant difference in age (*F*_2,113_=6.90, *P*=.001, ω^2^=.09) when comparing the 3 groups. A posthoc Tukey honestly significant difference test showed a significant difference (*P*=.001) between the RCT intervention and normative groups (mean difference 0.41) (Table 2). The intervention and control groups were combined as the alcohol-exposed group for subsequent analyses. The children in the alcohol-exposed group (n=76) had been, on average, exposed to 5.51 standard units of alcohol (SE 0.67) on at least 1 occasion during gestation (median 7.5 units).

**Table 2:** Characteristics of children and their mothers included in the randomized controlled trial.

Characteristic				Alcohol exposure (n=76)					No alcohol exposure (n=40)			*P* value
			RCT^a^ intervention (n=39)		RCT control (n=37)							
**Children**												
	Age (years), mean (SD)		4.72 (0.50)		4.83 (0.54)		5.14 (0.44)			.001		
	**Gender, n**									.92		
		Female	17		16		19					
		Male	22		21		21					
**Mother**												
	Age (years), mean (SD)		28.15 (5.32)		30.41 (5.85)		31.17 (6.18)			.08		
	Number of living children, mean (SD)		1.87 (0.95)		2.35 (1.27)		2.15 (1.21)			.19		
	Years of schooling, mean (SD)^b^		10.67 (1.69)		11.03 (1.78)		10.71 (2.07)			.66		
	Gravidity, mean (SD)		2.21 (1.10)		2.51 (1.40)		2.45(1.47)			.57		
	Monthly household income (ZAR)^c^, mean (SD)^d^		4953.24 (4619.59)		4610.56 (3375.28)		5474.86 (6233.52)			.75		
	**Pregnancy with participant planned, n (%)**									.27		
		Yes	11 (28)		12 (32)		18 (45)					
		No	28 (72)		25 (68)		22 (55)					
	**Received South African Social Security Agency grants, n (%)**											.22
		Yes	30 (76)		29 (78)		25 (62)					
		No	9 (24)		8 (22)		15 (38)					
	**Currently employed^e^, n (%)**									.59		
		Yes	26 (68)		26 (70)		24 (60)					
		No	12 (32)		11 (30)		16 (40)					

^a^RCT: randomized controlled trial.

^b^Missing data: n=1, n=4, and n=2 in the intervention, control, and no exposure groups, respectively.

^c^ZAR: South African Rand; an approximate exchange rate of ZAR 1 to US $0.07 is applicable at the time of publication.

^d^Missing data: n=3, n=4, and n=3 in the intervention, control, and no exposure groups, respectively.

^e^Missing data: n=2 and n=3 in the intervention and control groups, respectively.

There was no significant difference in monthly household income (mean difference ZAR 690.62, 95% CI −1259.95 to 2641.18, *t*_108_=0.702, *P*=.48), South African Social Security Agency grant receipt (alcohol-exposed: 59/76, 78%; nonexposed: 25/40, 62%; χ_1_^2^=3.00, *P*=.08), or caregiver unemployment (alcohol-exposed: 24/76, 30%; nonexposed: 16/40, 40%; χ_1_^2^=1.01, *P*=.32) between the groups.

Alcohol-exposed (combined intervention and control groups) and nonexposed group mean scaled scores for all NEPSY-II subtests (except statue) fell into the borderline performance category (between the 11th and 25th percentiles) or lower. Children in the alcohol exposed group and the nonexposed group performed below the expected level (at or below the tenth percentile) for comprehension of instructions (means 4.97 and 5.37, respectively), narrative memory recall (means 5.10 and 5.14, respectively), and sentence repetition (means 6.24 and 5.46, respectively) subtests. Performance on the statue subtest was at the expected level for both the alcohol exposed (mean 10.68) and nonexposed (mean 10.26) groups.

Some participants were unable to successfully complete all subtests in the chosen battery (based on the NEPSY-II guidelines for discontinuation [35]). The multivariate analysis of variance included data from 107 children. The relationship between alcohol exposure and NEPSY-II subtest scores was not significant (*V*=0.081, *F*_8,98_=1.073, *P*=.39). Posthoc univariate analyses on the subtest scores by group membership also revealed no significant differences (Table 3). The greatest variability in scores was found in the memory for designs (spatial) subtest (partial eta squared 0.033).

**Table 3:** Between-participant effects with alcohol exposure as the predictor variable.

Dependent variable		Type III sum of squares	*F* test (*df1*)	Partial eta squared	*P* value
**Language**					
	Comprehension of instructions	3.753	0.560 (1)	0.005	.46
**Memory and learning**					
	Memory for designs content	3.263	0.482 (1)	0.005	.49
	Memory for designs spatial	20.849	3.566 (1)	0.033	.06
	Memory for designs	6.172	2.076 (1)	0.019	.15
	Narrative memory free and cued	0.049	0.008 (1)	0.000	.93
	Narrative memory and recognition contrasted	4.636	0.427 (1)	0.004	.52
	Sentence repetition	14.291	2.625 (1)	0.024	.11
**Attention and executive function**					
	Statue	4.222	0.518 (1)	0.005	.47

## Discussion

### General

Performance on the NEPSY-II was poor, regardless of alcohol exposure. The data did not show any significant differences between the alcohol-exposed and nonexposed groups (*P*=.39).

### Group Comparison

The differences in demographic characteristics of the mothers of children in the RCT intervention, RCT control, and nonexposed groups were not significant (age: *P*=.08; living children: *P*=.19; years of schooling: *P*=.66; gravidity: *P*=57), which decreases the possibility that there is a significant environmental variable other than alcohol exposure that can explain any observed differences. The difference in the age between the children in the RCT intervention group and the nonexposed group was significant, with a medium effect size of 0.09; the nonexposed group was an average of 5 months (mean difference 0.41 years) older than the intervention group. The impact of this is minimized by using the scaled scores from the NEPSY-II, therefore although significant, this difference was not concerning.

### NEPSY-II Outcomes

The lack of difference between the alcohol-exposed and nonexposed groups was surprising given the strong associations that have been shown between alcohol use and deficits in executive function [10,42,43]. The average number of standard units of alcohol to which children were exposed exceeded the level defined as binge drinking (4 units of alcohol in 1 sitting [44]), and overall exposure to alcohol during pregnancy is based on a minimum estimate of exposure during pregnancy. The majority of women in the alcohol-exposed group used alcohol in a pattern associated with the highest risk of harm to pregnancy (average consumption was 5.51 units on one occasion).

NEPSY-II performance was similar for the exposed and nonexposed children. The finding of borderline scores on the NEPSY-II subtests for the entire group is unexpected and concerning. We hypothesized that the alcohol-exposed group would generally perform below expectation, and this was indeed the case. The nonexposed group, barring other causes of poor development, were expected to perform at age-appropriate levels. This did not prove to be the case, with no differences found between children in the alcohol-exposed group and nonexposed group.

It is possible that development of children in the alcohol-exposed group has not been impacted, with development occurring at the same rate as their peers. This does not imply that development of children in the alcohol-exposed groups will remain on par with that of their peers. It has been shown that the developmental trajectory of children with prenatal alcohol-exposure diverges from expected norms as they become older, which makes diagnosis and identification easier as time passes [45]. The young age of this cohort may therefore be a confounding factor.

As participants were recruited from underresourced and low–socioeconomic status areas, overall low performance may be linked to exposure to adverse childhood experiences. Adverse childhood experiences have a detrimental impact on cognitive development, social development, and mental health [46,47]. The impact on cognitive development overlaps with the impact on areas of development that we would expect from prenatal alcohol exposure [48]. It is important to note that, although adverse childhood experience measures frequently focus on abuse, neglect, parental separation, and exposure to criminality [49-51], this list is not exhaustive [49]. It is well established that poverty has a negative impact on cognitive assessments [52,53].

With 84 out of 116 (72%) households relying upon social grants, which amounts to only ZAR 410.00 (approximately US $27) each month per child for child support, these data indicate there are high levels of poverty and adversity. There is little that differentiates the alcohol exposed and unexposed groups, except for mothers’ reported alcohol use. The Saldanha Bay municipal area also experiences problems with violence and crime [31], and because adversity can be defined as exposure to a combination of deprivation and threat [54], it is likely that a significant number of participants in both groups would fit the definition of having had exposure to childhood adversity.

It is also possible that NEPSY-II cannot detect differences between groups. It may be that the NEPSY-II is not sensitive enough to detect the difference in executive function between alcohol-exposed and nonexposed groups. There are, however, no other assessments used in research on executive function or fetal alcohol spectrum disorder that have more suitable norms or that are more culturally appropriate for a South African context. Measuring executive function in these age groups is complicated by the way in which executive function develops; different aspects of executive function develop at different times and at different rates. Attention control, for example, develops and matures before cognitive flexibility (which is related to working memory and inhibition). This development is consistent with spurts of growth and development in the frontal lobe [55]. Major developmental periods continue until approximately 13 years of age, but further improvements in executive function continue due to myelination of prefrontal connections into adolescence [55]. In the NEPSY-II, more subtests are available to test executive function from 6 years of age [35], and the combination of fewer subtests and the variable nature of executive function development can also mask potential developmental differences. This would, however, hold true for other available assessments as well.

We must also acknowledge that there may be children exposed to alcohol in the normative group. Although selection was based on a confirmation of prenatal alcohol exposure, some mothers may have been reluctant to admit drinking or they may have misreported the amount that they drank during pregnancy because alcohol use during pregnancy is heavily stigmatized [56].

### Limitations

One of the study limitations is the lack of local norms for the NEPSY-II. The lack of local norms for neurodevelopmental assessments is an acknowledged problem in South Africa [38]. Language and culture bias may lead to a misrepresentation of actual cognitive abilities. These limitations were considered during the design phase of the study.

Another limitation is that we relied upon self-reporting of alcohol use. Underreporting of alcohol use was a concern due to possible reluctance on the part of the mother to admit to alcohol use. Mothers may also have found it difficult to recall alcohol use 5 years earlier. Some of the children in the nonexposed group may in fact have been exposed to alcohol. Inclusion due to alcohol use was based on recall of an average drinking session during pregnancy. Participants in the nonexposed group may, therefore, have been exposed to alcohol over a longer period of time but their reported alcohol use placed them in the nonexposed category because the level of exposure did not meet the threshold indicated in the fetal alcohol spectrum disorder diagnostic criteria [1].

Due to the size of the ECD centers, it was not possible to reach the total sample size in a single area or ECD center. Although the ECD centers were located in similar communities, there were still differences between centers that could have impacted on the performance of the children on the psychometric assessments. Some of these factors could include the number of children per ECD practitioner or the availability of toys and equipment for stimulation. It is well established that poverty has a negative impact on cognitive assessments [52,53].

### Conclusions

We compared the performance of children exposed and not exposed to alcohol in utero on measures of executive function. We expected that alcohol exposure would have a measurable negative impact, but in our sample, there was none. This highlights that developmental delays are widely prevalent in resource poor and low–socioeconomic status communities in South Africa. As developmental delays form part of the diagnostic criteria of fetal alcohol spectrum disorder, this study also shows that caution should be used when interpreting normed scores. It is possible that a borderline or below average score may not necessarily support a diagnosis of fetal alcohol spectrum disorder as there are clearly other possible causes of poor development that must be excluded.

This paper also identified important avenues for further research. The lack of difference between alcohol-exposed and nonexposed groups needs to be further explored. Does this lack of difference remain as participants age? What is the prevalence of adverse childhood experiences and to what extent do adverse childhood experiences explain the lack of difference between the groups? Overall, this paper adds to the understanding that alcohol exposure in utero and its sequelae are only part of the possible developmental challenges faced by children in South African communities.

## Abbreviations

ECD: early childhood development
RCT: randomized controlled trial
ZAR: South African Rand
